# Prognostic significance of Cytokeratin 20-positive lymph node vascular endothelial growth factor A mRNA and chromodomain helicase DNA binding protein 4 in pN0 colorectal cancer patients

**DOI:** 10.18632/oncotarget.23424

**Published:** 2017-12-19

**Authors:** Sze Chuen Cesar Wong, Moon Tong Cheung, Lewis Lai Yin Luk, Vivian Ha Man Lee, Pak Tat Chan, Hin Fung Andy Tsang, Evelyn Yin Kwan Wong, Vivian Weiwen Xue, Amanda Kit Ching Chan, John Kwok Cheung Chan

**Affiliations:** ^1^ Department of Health Technology and Informatics, Hong Kong Polytechnic University, Hong Kong, China; ^2^ Department of Surgery, Queen Elizabeth Hospital, Hong Kong, China; ^3^ Department of Pathology, Queen Elizabeth Hospital, Hong Kong, China

**Keywords:** prognostic significance, cytokeratin 20 lymph node, vascular endothelial growth factor A mRNA, chromodomain helicase DNA binding protein 4 mRNA

## Abstract

**BACKGROUND:**

Cytokeratin 20-positive cells in lymph nodes from pN0 colorectal cancer (CRC) patients were detected previously by us. The aims of this study were to investigate which tumor metastasis-related genes were involved and their potential clinical significance.

**RESULTS:**

Fourteen of 84 (17%) genes were differentially expressed by at least 2-fold. Among them, 10 genes were up-regulated whereas 4 genes were down-regulated. Those differential expressed genes were validated in the second cohort of specimens. Follow-up analysis for 60 months showed that patients with lymph node *vascular endothelial growth factor A (VEGF-A)* mRNA and *chromodomain helicase DNA binding protein 4 (CHD4)* mRNA expression higher than the median copies had significantly shorter time to recurrence than those with lower than the median copies. Multivariate analysis showed that *VEGF-A* mRNA, *CHD4* mRNA and lymphatic vessel involvement were independent prognostic factors for disease recurrence.

**CONCLUSIONS:**

*VEGF-A* mRNA and *CHD4* mRNA were up-regulated in CK20-positive pN0 lymph nodes and they may have prognostic significance in pN0 CRC patients.

**METHODS:**

Two cohorts of lymph node specimens from pN0 CRC patients of each with and without CK20-positive cells were recruited. In the first cohort, tumor metastasis genes were profiled using gene expression arrays. Differential expressed genes were validated in the second cohort. Moreover, their prognostic significance was examined by following-up the second cohort of patients with CK20-positive cells for 60 months and all histopathological findings were correlated to recurrence.

## INTRODUCTION

Colorectal cancer (CRC) is the second leading cause of cancer-related deaths in the Western world [1]. Despite curative surgery, approximately 40% of patients still experience disease relapse leading to morbidity and eventual mortality [2]. The main cause of death among patients with CRC is metastasis that can occur in regional lymph nodes (LNs) [3] or via blood to other distant organs [4]. Therefore, sensitive methods of detecting malignant cells in the LNs and blood may improve the prognostication of patients with pathologically-determined node-negative (pN0) CRC. With the impact of technological advancements like immunohistochemical (IHC) staining and quantitative reverse transcription-polymerase chain reaction (QRT-PCR) in the past decades, the traditional pathologic classifications used by several generations of pathologists that are only dependent on traditional haematoxylin and eosin staining have been challenged [5–7]. Cytokeratin 20 (CK20) is a low-molecular-weight CK with restricted expression in the gastrointestinal epithelium, urothelium, and Merkel cells [8]. This profile is maintained in the malignant tumors of these cells. As conflicting results have been reported on the prognostic value of CK20-positive cells in pN0 CRC [9, 10], we used a non-biotin polymer detection system to detect CK20-positive cells in the LNs of pN0 CRC patients and found that 29 out of 56 (52%) LN specimens had CK20-positive cells (Range: 1–35) [11]. At 12-month follow-up, 4 patients (4/29 = 14%) developed metastases to liver, lung and bone [11]. That study provided evidence that CK20-positive cells could be found in the LNs of pN0 CRC patients. In this study, we continued to investigate which tumor metastasis-related genes were involved in this micrometastatic pathway. Differential expressed genes detected were validated in another cohort of pN0 CRC patients followed by examining their prognostic significance. The information obtained would be very useful for us to understand the biology of CK20-related micrometastatic pathway in the pN0 CRC and its potential clinical applications.

## RESULTS

### Anti-CK 20 IHC staining in the first cohort of pN0 LN specimens from 23 CRC patients of each with and without CK20-positive cells

**Figure 1:**
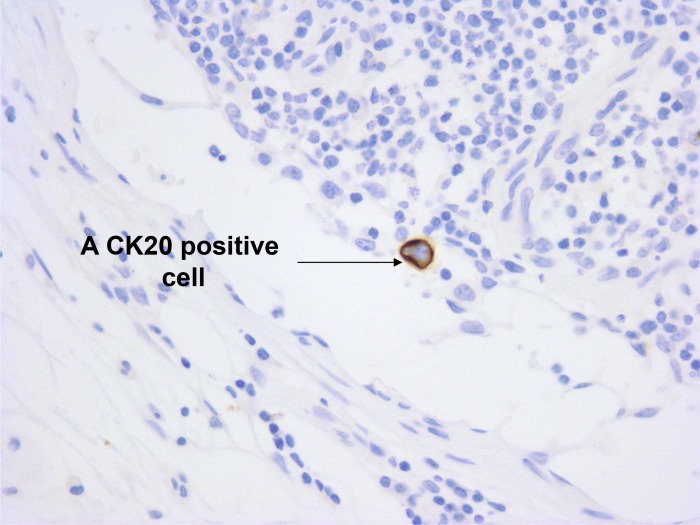
Immunostaining for CK20 in a pN0 LN A CK20 positive cell was located within the sinuses of the LN. Original magnification X 400.

**Figure 2:**
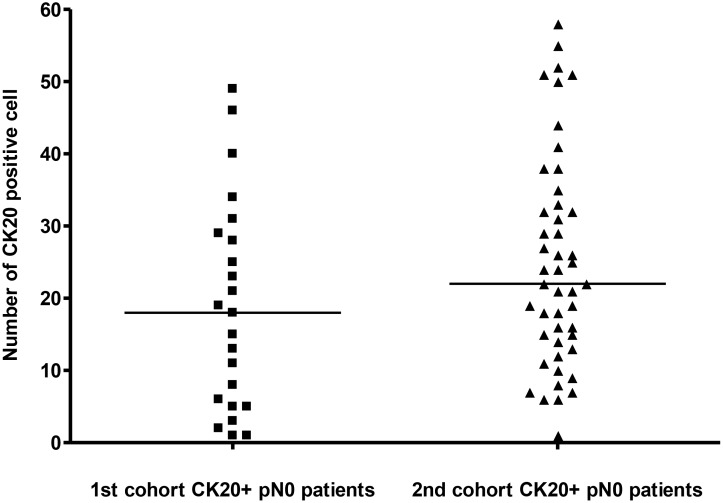
The number of CK20 positive cell detected in LN from the 1st and the 2nd cohorts of pN0 CRC patients The median in each group of subjects was indicated by a black horizontal line.

CK20-positive cells were frequently arranged in isolation or less commonly as tiny clusters measuring less than 0.1 mm. They were found within sinuses, often in the subcapsular region. They showed strong membrane positivity for CK20. Those cells were interpreted as carcinoma cells since their nuclei showed nuclear enlargement, but usually not as prominent as the cells in the main tumor (Figure 1). The number of CK20-positive cell was the same for both stained sections of each specimen and the total numbers of CK20-positive cell detected in the LNs from each patient were shown in Figure 2 (1st cohort: Range = 1 to 49; median = 18). No CK20-positive cell was found in the control group.

### Tumor metastasis PCR arrays

**Figure 3:**
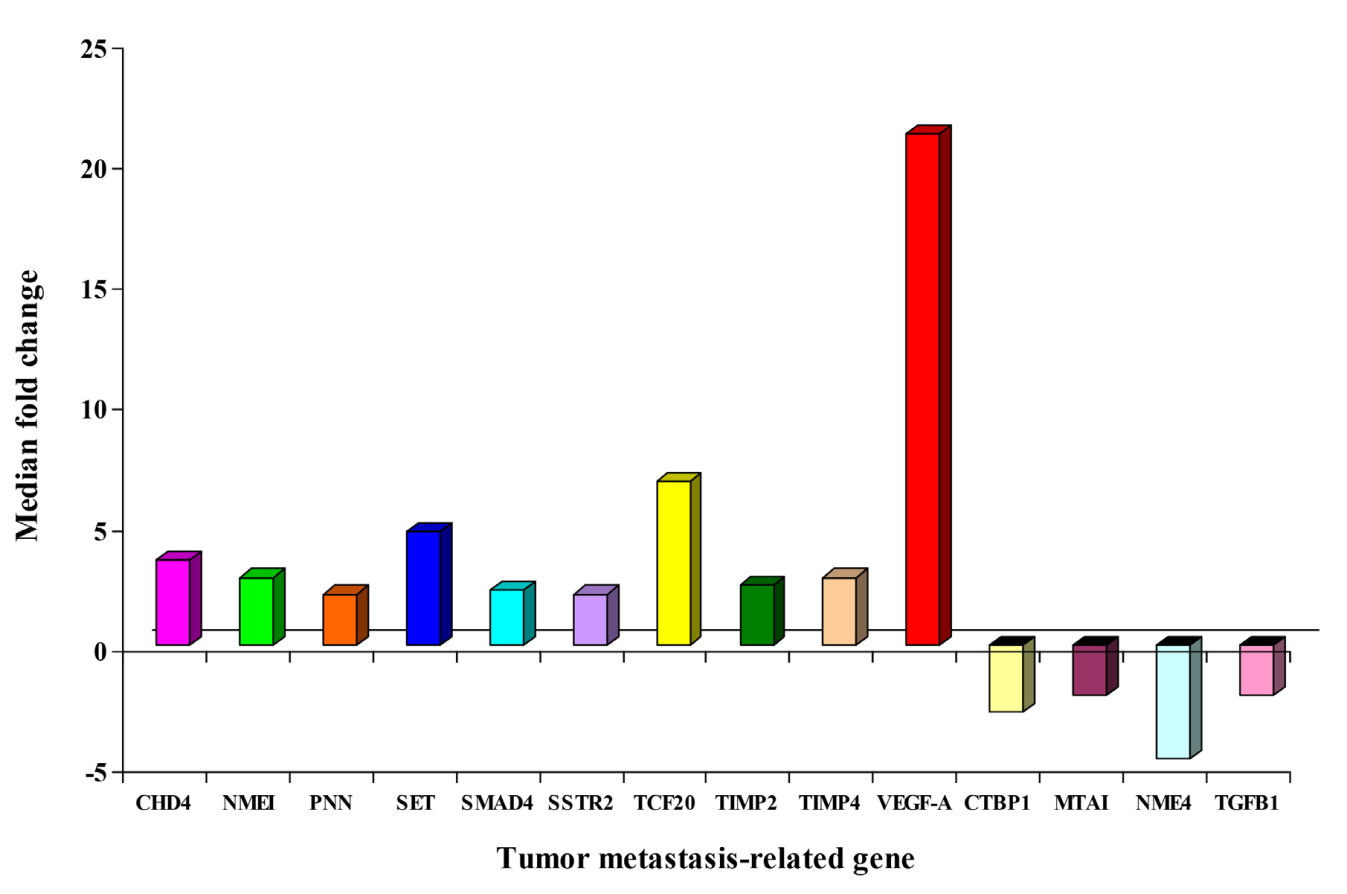
Median fold change of differential expressed genes from the 1st cohort of pN0 CRC patients

The no template control and no RT control of each PCR array were negative. Fourteen of 84 (17%) genes were differentially expressed by at least 2-fold in CK20-positive specimens when compared with specimens without any CK20-positive cell. Among them, the expression of *chromodomain helicase DNA binding protein 4 (CHD4), non-metastatic cells 1 (NME1), pinin (PNN), SET translocation (SET), SMAD family member 4 (SMAD4), somatostatin receptor 2 (SSTR2), transcription factor 20 (TCF20), TIMP metallopeptidase inhibitor 2 (TIMP2), TIMP metallopeptidase inhibitor 4 (TIMP4), vascular endothelial growth factor A (VEGF-A),* were up-regulated and that of *C-terminal binding protein 1 (CTBP1), metastasis associated 1 (MTA1), non-metastatic cells 4 (NME4), transforming growth factor beta-1 (TGFB1),* were down-regulated. The median fold change for each differential expressed gene was shown in Figure 3. *VEGF-A* gene and *TCF20* gene were selected for validation in the second cohort of specimens because they had the highest median fold-changes of 21.2 and 6.8, respectively.

### Validation of VEGF-A mRNA and TCF20 mRNA in the second cohort of pN0 LN specimens from 47 CRC patients of each with and without CK20-positive cells

### Anti-CK 20 IHC staining

The number of CK20-positive cell was the same for both stained sections of each specimen. The total number of CK20-positive cell detected in the LNs from each patient was shown in Figure 2 (Range: 1 to 58; median: 22). No CK20-positive cell was found in the control group.

### QRT-PCR

**Figure 4:**
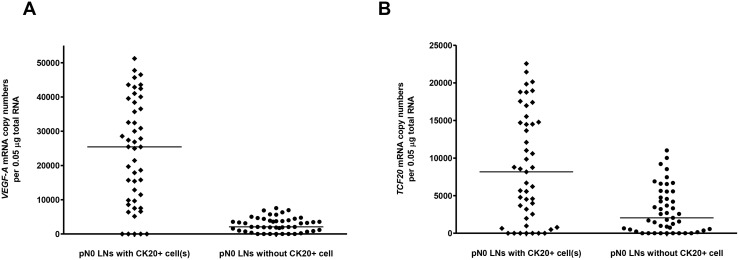
(**A**) *VEGF-A* mRNA and (**B**) *TCF20* mRNA copy numbers per 0.05 μg total RNA in CK20+ and CK20- LNs from the 2nd cohort of pN0 CRC patients. The median in each group of subjects was indicated by a black horizontal line.

*VEGF-A* mRNA was detected in 89.4% (42/47; range: 0 - 51240 copy numbers; median: 25431 copy numbers) and 85.1% (40/47; range: 0 - 7546 copy numbers; median: 2089 copy numbers) CK20-positive and CK20-negative pN0 LNs, respectively. *VEGF-A* mRNA expression in CK20-positive pN0 LNs was significantly higher than those in CK20-negative pN0 LNs (Figure 4A, *P* = 0.00007, Mann Whitney test). *TCF20* mRNA was detected in 85.1% (40/47, range: 0 - 22569 copy numbers; median: 8165 copy numbers) and 80.9% (38/47, range: 0 - 11021 copy numbers; median: 2047 copy numbers) CK20-positive and CK20- negative pN0 LNs, respectively. *TCF20* mRNA in CK20-positive pN0 LNs was significantly higher than those in CK20-negative pN0 LNs (Figure 4B, *P* = 0.0002, Mann Whitney test).

### Time to recurrence amongst the 47 CK20-positive pN0 CRC patients

**Figure 5:**
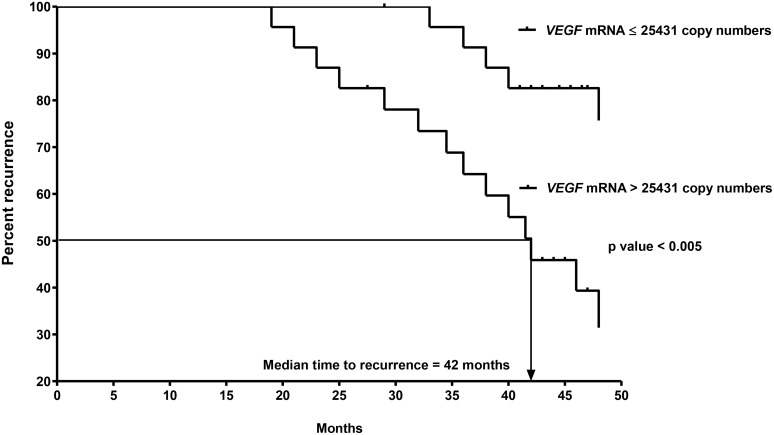
Kaplan-Meier curves of recurrence for the 2nd cohort of 47 CK20+ pN0 CRC patients after follow-up for 60 months based on *VEGF-A* mRNA copy numbers

**Figure 6:**
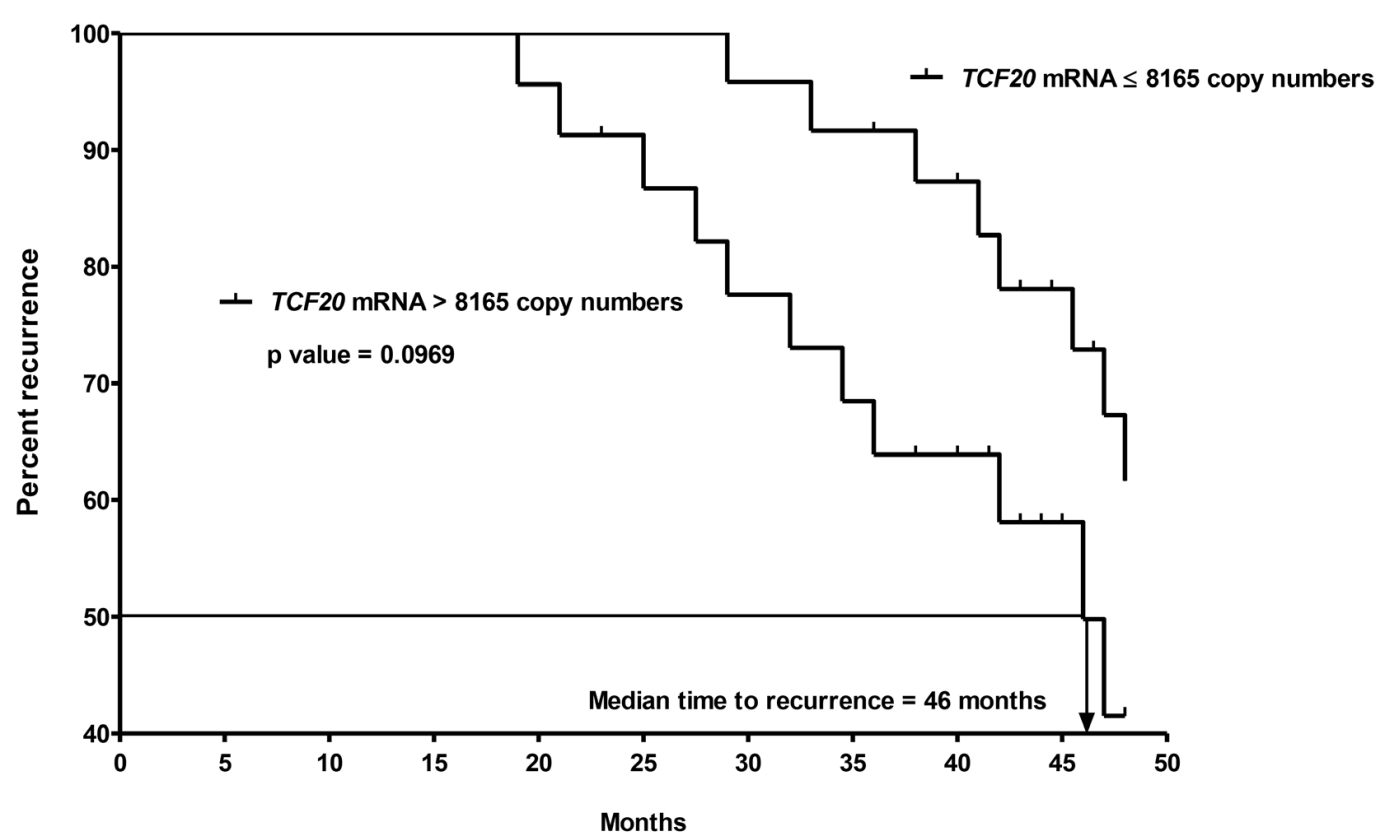
Kaplan-Meier curves of recurrence for the 2nd ohort of 47 CK20+ pN0 CRC patients after follow-up for 60 months based on *TCF20* mRNA copy numbers

All follow-up data was completed by July 2016 and 2 patients (2/47 = 4%) were lost to follow-up. Nineteen patients (19/47 = 40%) developed recurrent disease including 10 patients (10/47 = 21%) with local recurrence, 8 patients (8/47 = 17%) with distant metastases to liver, lung or bone and 1 patient (1/47 = 2%) with both local recurrence and distant metastases to liver. Amongst the 47 patients with CK20-positive pN0 CRC, 23 and 24 patients had *VEGF-A* mRNA concentrations that were > and ≤ to their median copy numbers, respectively. In terms of recurrence, 14 out of 23 patients who had *VEGF-A* mRNA concentration > median copies (25431) developed recurrence while only 5 out of 24 patients who had *VEGF-A* mRNA concentration ≤ median copies (25431) had recurrence. Using the median *VEGF-A* mRNA copy number as the cut-off point, the time to recurrence was significantly shorter for the 23 patients with *VEGF-A* mRNA concentration > 25431 copies (median time to recurrence = 42 months) than for the 24 patients with *VEGF-A* mRNA concentration ≤ 25431 copies (Figure 5, *P* = 0.0027, log-rank test; hazard ratio = 4.137; 95% confidence interval {CI} = 1.635 to 10.470). On the other hand, 11 out of 23 patients who had *TCF20* mRNA concentration > median copies (8165) developed recurrence when compared to 8 out of 24 patients who had *TCF20* mRNA concentration ≤ median copies (8165) had recurrence. Using the median *TCF20* mRNA copy number as the cut-off point, the time to recurrence was not significantly different between the 2 groups of patients. (Figure 6, *P* = 0.0969, log-rank test).

### Further validation of the remaining 12 genes in the second cohort of 31 PELS

**Figure 7:**
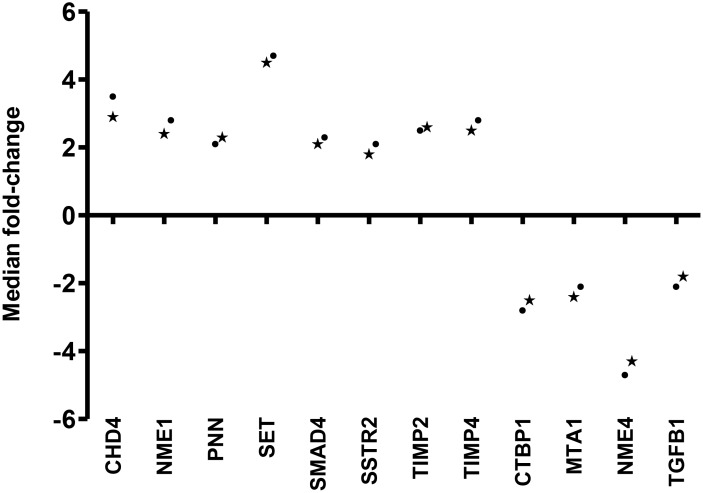
Median fold-change of 12 differential expressed genes derived from validation using QRT-PCR(*) and PCR array (·)

**Figure 8:**
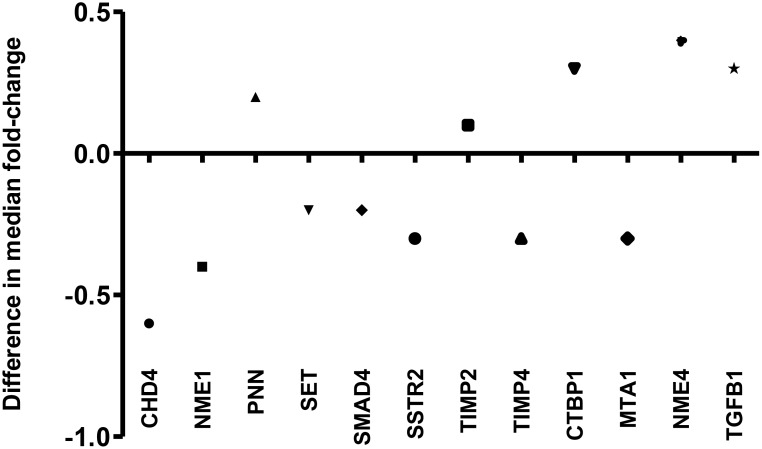
Difference in median fold-change of 12 differentiated genes derived from validation using QRT-PCR and PCR array

**Figure 9:**
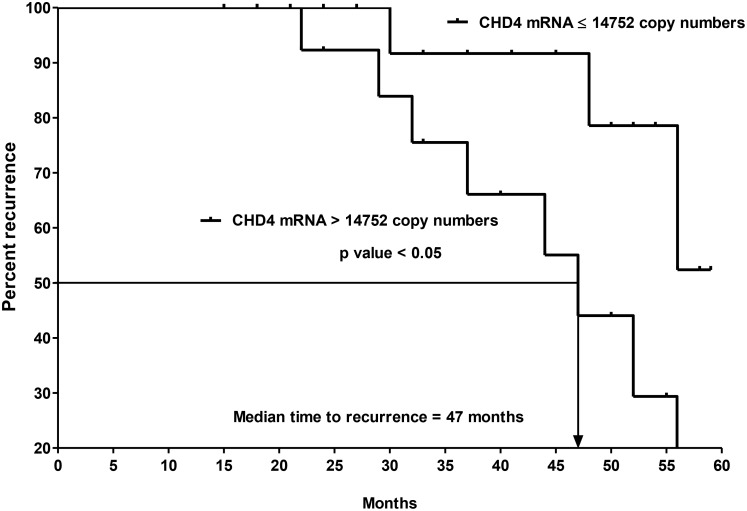
Kaplan-Meier curves of recurrence for the 2nd cohort of 31 CK20+ pN0 CRC patients after follow-up for 60 months based on *CHD4* mRNA copy numbers

The sensitivity of detection for the remaining 12 differential expressed genes was shown in Table 1. Moreover, the range of copy number of those genes in each group of specimens and their median fold-change were shown in Table 2. More detailed analysis showed that the median fold-change for each validated gene using QRT-PCR is close to that from PCR array (Figure 7) and their difference range from 0.1 to 0.6 (Figure 8). Out of 12 genes, only *CHD4* gene expression was found to correlate with disease recurrence. Amongst the 31 patients with CK20-positive pN0 CRC, 15 and 16 patients had *CHD4* mRNA concentrations that were > and ≤ to their median copy numbers, respectively. In terms of recurrence, 8 out of 15 patients who had *CHD4* mRNA concentration > median copies (14752) developed recurrence while only 3 out of 16 patients who had *CHD4* mRNA concentration ≤ median copies (14752) had recurrence. Using the median *CHD4* mRNA copy number as the cut-off point, the time to recurrence was significantly shorter for the 15 patients with *CHD-4* mRNA concentration > 14752 copies (median time to recurrence = 47 months) than for the 16 patients with *CHD4* mRNA concentration ≤ 14752 copies (Figure 9, *P* = 0.0303, log-rank test; hazard ratio = 3.826; 95% confidence interval {CI} = 1.136 to 12.88).

**Table 1:** Sensitivity of detection for the 12 validated differential expressed genes

mRNA genes	Detection in CK20-positive pN0 LNs (%)	Detection in CK20-negative pN0 LNs (%)
CHD4	28/31 (90.3)	23/31 (74.2)
NME1	26/31 (83.9)	24/31 (77.4)
PNN	29/31 (93.5)	24/31 (77.4)
SET	28/31 (90.3)	26/31 (83.9)
SMAD4	24/31 (77.4)	28/31 (90.3)
SSTR2	26/31 (83.9)	29/31 (93.6)
TIMP2	24/31 (77.4)	23/31 (74.2)
TIMP4	26/31 (83.9)	28/31 (90.3)
CTBP1	24/31 (77.4)	26/31 (83.9)
MTA1	28/31 (90.3)	28/31 (90.3)
NME4	26/31 (83.9)	28/31 (90.3)
TGFB1	24/31 (77.4)	24/31 (77.4)

**Table 2:** Copy number and median fold-change of 12 validated differential expressed genes

	CK20-positive pN0 LNs		CK20-negative pN0 LNs		
mRNA genes	Range of copy numbers	Median copy numbers	Range of copy numbers	Median copy numbers	Median fold-change
CHD4	0–31693	14752	0–24531	5084	2.9
NME1	0–42761	18241	0–19832	7558	2.4
PNN	0–38654	17694	0–13275	7691	2.3
SET	0–17876	7856	0–5639	1741	4.5
SMAD4	0–21347	8966	0–11476	4262	2.1
SSTR2	0–47843	21489	0–20175	11917	1.8
TIMP2	0–15781	5420	0–3877	2082	2.6
TIMP4	0–17841	4876	0–5642	1947	2.5
CTBP1	0–12743	3689	0–26874	9225	−2.5
MTA1	0–11057	2458	0–17563	5891	−2.4
NME4	0–8564	3589	0–43252	15421	−4.3
TGFB1	0–14896	5725	0–38766	10309	−1.8

### Multivariate analysis of clinicopathologic variables to recurrence

Multivariate analysis (Table 3) showed that *VEGF-A* mRNA, *CHD4* mRNA expression and lymphatic vessel involvement were associated with recurrence whereas *TCF20* mRNA expression, sex, age, pT-category and differentiation status were not. Detailed analyses showed that *VEGF-A* mRNA expression > 25431 copies was the strongest predictor of recurrence with a relative hazard of recurrence of 8.436 (*P* = 0.004).

**Table 3:** Multivariate regression for recurrence in pN0 CRC patients by Cox proportional hazards regression

Parameter	*P*-value	Relative hazard	95% CI for relative hazard
*VEGF-A* mRNA expression (>25431 copies vs ≤25431 copies)	0.004 (S)	8.436	5.682–16.190
CHD4 mRNA expression (>14752 copies vs ≤14752 copies)	0.021 (S)	5.174	2.617–12.731
*TCF20* mRNA expression (>8165 copies vs ≤8165 copies)	0.375 (NS)	–	–
Sex (Male vs female)	0.794 (NS)	–	–
Age (>71 years vs ≤71 years)	0.827 (NS)	–	–
pT-category, (T1 + T2 vs T3 + T4)	0.965 (NS)	–	–
Differentiation (well vs poor)	0.758 (NS)	–	–
Lymphatic vessel involvement (presence vs absence)	0.024 (S)	4.162	1.512–10.812

S = Significant; NS = Non-significant.

## DISCUSSION

This study is the first to compare the tumor metastasis-related genes expression between CK20-positive and CK20-negative pN0 LNs in CRC patients. Previous study has shown that CK20-positive circulating tumor cells have important clinical significance in patients with CRC [4]. Therefore, it is logical to hypothesize that CK20-positive cells in pN0 LNs will have clinical significance. The results generated from this study would improve our understanding in CK20-related micrometastasis in pN0 CRC patients. Fourteen differentially expressed tumor metastasis-related genes were discovered and the description for each of them was shown as below.

### Up-regulated genes

### CHD4

CHD4 proteins are a family of protein that possesses 2 N-terminal truncated chromodomains: a centrally located SNF2-like helicase motif, and a C-terminal DNA-binding domain [12–14]. By forming protein complexes with different partners, CHD4 may exert different functions including nucleosome remodeling, transcriptional activation [12], transcriptional repression, proliferation regulation and metastasis [13], and growth regulation [14].

### NME1

The protein product of the *NME1* gene is a nucleoside diphosphate (NDP) kinase which is responsible for the transfer of gamma-phosphates between di- and tri-phosphonucleosides in providing cells with high-energy nucleosides other than ATP [15]. By maintaining the homeostasis of cellular nucleoside di- and triphosphate composition, NME1 is involved in a variety of biological events such as tumor metastasis, cell proliferation, differentiation, motility, transcriptional regulation, development, senescence, and apoptosis [16].

### PNN

PNN is a cell adhesion-related molecule [17, 18]. By assembling to desmosome, it enhances cell junction formation, intercellular adhesion, and cytoplasmic intermediate filament formation [17, 18]. *PNN* is found to be up-regulated in a subset of melanomas [19]. The over-expression of *PNN* implies a compensatory mechanism which may function to circumvent the disrupted regulatory pathway of wild-type PNN protein [20].

### SET

The *SET* gene plays a key role in human acute undifferentiated leukemia by acting as a tumor promoter. The aberration involves chromosomal rearrangement as *SET* gene fuses to and activates the putative oncogene, *CAN*, so that a chimeric SET-CAN fusion protein is produced [21].

### SMAD4

The tumor suppressor gene *SMAD4* mediates the TGFb signaling pathway suppressing epithelial cell growth [22]. Although all adenomas and Dukes’ stage I colorectal adenocarcinomas expressed SMAD4 protein, progressive loss of SMAD4 protein from Dukes’ stage II to IV colorectal adenocarcinomas were observed [23]. This evidence suggests that inactivation of *SMAD4* is a late event in colorectal carcinogenesis.

### SSTR2

SSTR2 is one of the G-protein coupled receptors through which the multifunctional peptide hormone, somatostatin, regulates cell secretion and proliferation [24]. SSTR2 is a well defined prognostic and therapeutic target for neuroendocrine tumors [25].

### TCF20

*TCF20 gene*, located at human chromosome 22q13.3, encodes a regulator of gene expression [26]. *TCF20* is involved small cell lung cancer (SCLC) and advanced lung adenocarcinomas carcinogenesis and chemoresistance [27]. Moreover, *TCF20* expression can distinguish desmoid tumors from nodular fasciitis [28].

### TIMP2 and TIMP4

Matrix metalloproteinases promote tumor invasion and metastasis, regulating host defense mechanisms and normal cell function [29]. Metalloproteinases are inhibited by tissue inhibitors (TIMPs) which are secreted proteins. The role of TIMPs for the homeostasis of the extracellular matrix is critical and it may inhibit or stimulate tumorigenesis [30].

### VEGF-A

VEGF is one of the most important cytokine to induce angiogenesis so that tumor can grow and spread to other organs [31]. The ligands of the VEGF family include VEGF-A, VEGF-B, VEGF-C, VEGF-D, and VEGF-E. VEGF-A is the most abundantly expressed in CRC tissues and VEGF-A seems to be of greater value than total VEGF [32]. Previous study indicated that VEGF-A is induced by a keratinocyte growth factor in CRC cells and it can stimulate lymphangiogenesis indirectly by activating the VEGF-C/VEGF-D/VEGFR-3 signaling pathways [33].

### Down-regulated genes

### CTBP1

The main function of CTBP1 is to regulate gene expression patterns throughout development and in oncogenesis [34]. CTBP1 binds to adenomatous polyposis coli (APC) in both *Drosophila melanogaster* and in human cells [35]. Previous study showed that APC controls retinoic acid biosynthesis and intestinal differentiation, in part by negatively regulating the levels of CTBP1 [36].

### MTA1

The *MTA1* gene is a metastasis associated gene [37]. Previous studies showed that overexpression of *MTA1* gene was found in colorectal, gastric and small-intestinal carcinoid neoplasia [38]. In fact, *MTA1* gene expression correlates with tumor invasion, metastasis and that a high expression of *MTA1* mRNA may be a potential indicator for assessing the malignant potential of colorectal and gastric carcinomas [38].

### NME4

*NME4* encodes a mitochondrial protein which similar to *NME1*, has NDP kinase activity [39]. By catalyzing the transphosphorylation of GDP to GTP in the mitochondria, NME4 maintains the essential mitochondrial functions and constitutes an important link between energy metabolism and cellular regulation [39, 40].

### TGF-β1

TGF-β is a member of family consisting of 3 growth factors in mammalian cells and they are known as TGF-β1, TGF-β2 and TGF-β3 which are secreted in inactivated forms [41]. In carcinogenesis, TGF-β1 switches from tumor suppressor in the premalignant stages to pro-oncogene and pro-metastatic factor [42].

VEGF-A is a well-recognized protein to promote angiogenesis and metastasis in CRC [31–33]. The clinical relevance is further supported by a recent study that increased VEGF-A expression in primary CRC specimens was correlated to LN metastasis and worse prognosis in CRC patients [43]. Our findings are more significant because *VEGF-A* mRNA expression in pN0 LN not only may be involved in CK20-related micrometastasis pathway but also its expression can identify conventional histopathologically confirmed non-metastatic CRC patients with high risk of recurrence. When comparing to CK20-positive cells detection by IHC [11, 44], *VEGF-A* mRNA detection by RT-PCR is more powerful to predict CRC patients’ prognosis because currently the interpretation of IHC staining for CK20-positive cells are not standardized [44] and the number of CK20-positive cell is too small to reflect the clinical status of the patients. We are currently performing anti-VEGF-A IHC staining to examine whether VEGF-A protein will also be up-regulated in the pN0 LN. The results will be correlated to the clinical conditions of the patients in order to examine if LN VEGF-A protein expression will be helpful to select pN0 CRC patients for anti-VEGF-A therapy using Bevacizumab. This approach has an additional therapeutic effect by reducing VEGF-A stimulated lymphangiogenesis [45] which will improve patients’ prognosis because multivariate analysis showed that lymphatic vessel involvement is another significant factor contributing to recurrence. Despite this, 9 patients (9/23 = 39%) had higher than median LN *VEGF-A* copy number but they did not develop tumor recurrence. This phenomenon can be explained by the fact that the LN *VEGF-A* mRNA detected may not be biologically active to develop tumor recurrence or a longer period is required to develop recurrence. On the other hand, 3 patients (3/47 = 6%) developed recurrence even though they were CK20-negative in their pN0 LNs (data not shown) and this observation can show that CK20-related micrometastasis is only one of the major pathways which leads to recurrence and there may still have other pathway(s) promoting metastasis in pN0 CRC. In summary, our results have laid down a solid foundation for using *VEGF-A* mRNA as a potential prognostic factor and therapeutic target in pN0 CRC patients.

A recent paper has shown that *CHD4* helps to maintain DNA hypermethylation-associated transcriptional silencing of tumor suppressor genes. Moreover, the mRNA levels of *CHD4* are significantly higher in CRC than in normal colorectal tissues. This *CHD4* up-regulation has prognostic significance as it is an independent risk factor for highest recurrence rates and reduced survival time [14]. Our results can show that *CHD4* gene expression may further be involved in CK20-related micrometastasis. A large scale study is now undergoing to examine whether *CHD4* mRNA can work with *VEGF-A* mRNA to become a pair of prognostic markers in pN0 CRC patients.

The up-regulated expression of *TCF20* mRNA in CK20-positive pN0 LNs is another novel and interesting finding because there is no previous report on the involvement of *TCF20* mRNA in CRC. As *TCF20* shows extensive sequence identity to a mouse transcription factor which activates the expression of the *stromelysin-1* mRNA for tumor invasion and metastasis [26], therefore we will examine whether this function will be performed by TCF20 protein in human CRC cell lines.

### Conclusions and future perspectives

In summary, results generated from this study have shown that *VEGF-A* mRNA, *CHD4* mRNA were up-regulated in CK20-positive pN0 lymph nodes and they may have prognostic significance in pN0 CRC patients. Moreover, CK20-positive cell is not a marker of tumor metastasis in pN0 CRC patients. However the roles of *VEGF-A, CHD4* and *TCF20* genes in CK20-related micrometastasis are unknown, the expression of them in LNs from Tumor-Node-Metastasis (TNM) stage III and IV CRC patients would be studied in order to know whether this micrometastasis pathway is preserved in metastatic LNs. Moreover, we will establish cut-off values of LN *VEGF-A* mRNA and *CHD4* mRNA copy numbers so as to select pN0 CRC patients for adjuvant therapy after confirming those results. Finally, the functional significance of those 3 genes will also be explored in human CRC cell lines. However, the major pitfall of this study is the small patient sample size and a larger scale of study is necessary to validate our findings. Technically, the number of LN specimens per patient used is too large for routine clinical application and optimization to select LN specimens according to their size, location or distance to the primary tumor is essential. Moreover, the detection of CK20-positive cells under microscope is time-consuming and a fully automated imaging system should be used to scan for CK20-positive cells.

Cancer metastasis is a complicated process which involves multiple pathways [46] and predicting the risk of recurrence is essential to improve patient management. The results from this study provide strong evidence that the combination of quantitative PCR arrays and QRT-PCR are able to discover and validate markers which can improve prognosis in pN0 CRC patients by re-classifying the TNM stage I and II CRC patients according to their risk of recurrence. However, molecular analysis will supplement but cannot replace the most classical but less expensive, internationally validated procedures of the “traditional” surgical pathology. Nevertheless, before the implementation of these molecular tests as a routine practice, the standardization of various important parameters including LN sampling methods, criteria and scanning method of CK20-positive cells, molecular protocols, and clinical follow-up standards has to be performed so that we can verify the prognostic impact of our findings. In future, our ultimate aim is to identify the high risk subgroup of pN0 CRC patients so that they can be closely monitored or to receive adjuvant chemotherapy.

## MATERIALS AND METHODS

### Patients and tissues

**Figure 10:**
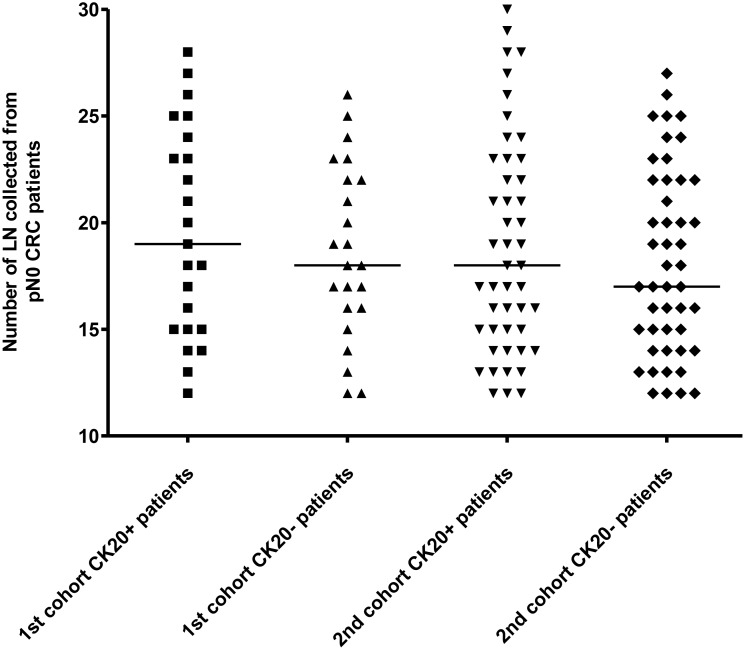
The number of LN examined from the 1st and the 2nd cohort of pN0 CRC patients The median in each group of subjects was indicated by a black horizontal line.

In the first cohort, 23 pN0 CRC patients each with and without CK20-positive cells were recruited whereas in the second cohort, 47 pN0 CRC patients each with and without CK20-positive cells were collected. All the paraffin-embedded LN specimens (PELS) were recruited in the Department of Pathology, Queen Elizabeth Hospital (QEH), Hong Kong Special Administrative Region (HKSAR). As it is recommended by the American Joint Committee on Cancer (AJCC) and College of American Pathologists that a minimum of 12 LNs be reviewed for accurate staging [47], therefore the numbers of LN examined in the tested and control groups from the first and second cohort were shown in Figure 10 (1st cohort: CK20-positive LNs: Range: 12 to 28, median: 19; CK20-negative LNs: 12 to 26, median: 18; 2nd cohort: CK20-positive LNs: Range: 12 to 30, median: 18; CK20-negative LNs: Range: 12 to 27, median: 17). Macrodissection was performed on both cohorts of patient specimens. The study was approved by the Clinical Research Ethics Committee of QEH, HKSAR. The profiles of patients for both cohorts were shown in Table 4 and no patient had received pre-operative chemotherapy or radiotherapy.

**Table 4:** Patients profiles

pN0 LN specimens	p-TNM stage	pT Value	Gender	Age range (Years)	Location of tumor	Histological grade	Range of tumor size [Transverse dimension] (cm)	Lymphovascular invasion
1st cohort	Stage I (5/46, 11%) Stage II (41/46, 89%)	T1 (1/46, 2%) T2 (4/46, 9%) T3 (36/46, 78%) T4 (5/46, 11%)	Male: 28/46, 61% Female: 18/46, 39%	33-87 (Mean age: 69)	Ascending colon (12/46, 26%) Descending colon (9/46, 20%) Sigmoid colon (14/46, 30%) Rectum (11/46, 24%)	Adenocarcinoma: (1) Well differentiated 2/46, 4% (2) Moderately differentiated 40/46, 87% (3) Poorly differentiated 4/46, 9%	2.0–12.5 (Mean: 4.5 )	Present: 10/46, 22% Absent: 36/46, 78%
2nd cohort	Stage I (9/94, 10%) Stage II (85/94, 90%)	T1 (2/94, 2%) T2 (10/94, 11%) T3 (74/94, 79%) T4 (8/94, 8%)	Male: 55/94, 59% Female: 39/94, 41%	31-92 (Mean age: 71)	Ascending colon (22/94, 23%) Descending colon (19/94, 20%) Sigmoid colon (29/94, 31%) Rectum (24/94, 26%)	Adenocarcinoma: (1) Well differentiated 4/94, 4% (2) Moderately differentiated 80/94, 85% (3) Poorly differentiated 10/94, 11%	1.8–13.6 (Mean: 4.9)	Present: 20/94, 21% Absent: 74/94, 79%

### Anti-CK20 IHC staining and evaluation

Two serial tissue sections (4 μm thick per section) were cut for each CK20 positive and CK20 negative PELS and IHC staining was performed according to a protocol as shown in our previous study [11]. The criteria for a CK20-positive cell inside a LN are based on the AJCC which has defined “isolated tumor cells” as lesions smaller than 0.2 mm [47]. The whole stained slides were evaluated under light microscope at magnification X 400 by 2 independent pathologists without knowledge of clinical outcomes and in the case of disagreement, consensus was reached after thorough discussion and slides examination using a multi-headed microscope. All slides were scored and expressed as the average number of CK20-positive cell per patient. Slides with a CK20-positive cell detected exclusively outside the LN capsule were not selected for molecular analysis.

### Macrodissection

Thirty serial sections (5 μm thick per section) of each CK20 positive and CK20 negative PELS as detected from IHC staining were cut. Microtome was cleaned with xylene before sectioning of each specimen in order to avoid any tissue carryover. Each section was mounted on a superfrost slide. The unstained sections of each specimen were deparaffinized with xylene followed by absolute alcohol. Selected areas on each slide were circled by comparing with a reference IHC stained slide of the same tissue section. Circled areas on each slide were filled with buffer ATL (Qiagen, Hilden, Germany) followed by scrapping using a new scalpel for each tissue specimen. The scrapped tissues were then transferred into an RNase-free microcentrifuge tube and the final volume was made up to 180 μl using buffer ATL. DNA extraction was performed according to instructions of a QIAamp DNA paraffin embedded tissue kit (Qiagen).

### RNA extraction

Total RNA was extracted from macrodissected paraffin-embedded sections per LN specimen. The procedures were briefly described as follows: The sections were dewaxed by standard xylene and ethanol wash. The harvested pellet was speed vac to complete dryness. Protease digestion began by suspending the pellet in 400 μL of proteinase K (PK) digestion buffer containing 20 mM Tris, pH 7.5, 20 mM EDTA and 1% SDS supplemented with 20 μL of 20 mg/mL PK (Roche Diagnostics Corporation, USA) at 56 °C for at least 24 hours until the pellet was completely dissolved. The supernatant from all LN specimens for each patient was pooled together and total RNA was extracted using the QIAamp Viral RNA Mini Kit (Cat. no. 52904, Qiagen) followed by DNase treatment (Cat. no. 18068-015, Invitrogen, Carlasbad, CA, USA) for 30 minutes. Extracted total RNA was then purified using the RNeasy Mini Kit (Cat. no. 74106, Qiagen) according to the manufacturer's protocol. Residue genomic DNA contamination was verified by a TaqMan quantitative assay (Cat. no. 401846, Applied Biosystems, Foster City, CA, USA) without RT for beta-actin DNA.

### RNA quality control

RNA concentration was determined by measuring the absorbance at 260 nm using a spectrophotometer (DU650, Beckman Coulter, Fullerton, CA, USA). Each sample was measured for 3 times and an average reading was obtained for the calculation of RNA concentration. Sample purities were determined by A260/A280 and A260/A230 ratios. Samples of A260/A280 ratio between 1.8 and 2.2 and of A260/A230 ratio greater than 1.7 were used.

### Tumor metastasis PCR arrays

Gene expressions were studied by tumor metastasis PCR arrays (SuperArray: RT^2^ Profiler^TM^ PCR Array System) according to the manufacturer's protocol using 7500 Real Time PCR System (Applied Biosystems). Briefly, 2 μg of total RNA of each patient was reverse transcribed using the ReactionReady^TM^ First Strand Synthesis Kit (SuperArray Cat. no. C-01, SABiosciences, Frederick, USA) and the reaction mix was aliquoted into the wells of the array containing pre-dispensed gene-specific primer sets. Each array included 5 housekeeping genes and 2 negative controls.

### Data analysis

The fold-change for each gene between patient specimens with and without CK20-positive cells was calculated by the ΔΔCt method and the average Ct value of all 5 housekeeping genes was used for normalization according to manufacturer's instructions. The median of all fold-changes obtained was calculated for each transcript.

### Validation of vascular endothelial growth factor A (VEGF-A) mRNA and transcription factor 20 (TCF20) mRNA in the second cohort of PELS

TaqMan gene expression assays of specific primers and MGB probes were purchased from Applied Biosystems for the detection of *VEGF-A* mRNA (Hs00900054_m1) and *TCF20* mRNA (Hs00390028_m1). QRT-PCR was performed in a reaction volume of 50 μL using Taqman Universal PCR Master Mix (Cat. no. 4304437; Applied Biosystems), and 10 μL of cDNA was used for each reaction. The standard protocol of the 7500 Real Time PCR System (Applied Biosystems) was used for both genes. Each batch of reaction included positive and negative controls and the copy numbers of *VEGF-A* mRNA and *TCF20* mRNA for each sample was calculated from standards prepared by serial dilutions of *VEGF-A* mRNA and *TCF20* mRNA-cloned plasmids. Duplicate tests were performed and the average was calculated for each sample.

### Further validation of the remaining 12 genes in the second cohort of PELS

Among 47 pN0 CRC patients each with and without CK20-positive cells, only 31 specimens of each group had sufficient DNA for the validation of the remaining 12 mRNA genes. TaqMan gene expression assays of specific primers and MGB probes were purchased from Applied Biosystems for the detection of 1) *CHD4* (Hs00172349_m1), 2) *NME1* Hs00264824_m1, 3) *PNN* Hs00170192_m1, 4) *SET* Hs04276680_m1, 5) *SMAD4* Hs00929647_m1, 6) *SSTR2* Hs00990356_m1, 7) *TIMP2* Hs00234278_m1, 8) *TIMP4* Hs00162784_m1, 9) *CTBP1* Hs00972284_m1, 10) *MTA1* Hs00950776_m1, 11) *NME4* Hs00359037_m1 and 12) *TGFB1* Hs00998133_m1. The same protocol as that to validate *VEGF-A* mRNA and *TCF20* mRNA was used.

### Follow-up of 47 CK20-positive pN0 CRC patients for recurrence

Forty-seven CK20-positive pN0 CRC patients from the second cohort were follow-up at periodic intervals in the Department of Surgery according to a standardized protocol. The follow-up period was 60 months from their respective diagnosis. Recurrent curves were plotted using the median copy numbers of *VEGF-A* mRNA, *TCF20* mRNA and *CHD4* mRNA as their respective cut-off points.

### Statistical analysis

The difference in copy numbers of the *VEGF-A* mRNA, *TCF20* mRNA and *CHD4* mRNA between CK20- positive pN0 LN and CK20-negative pN0 LN were analyzed by the Mann–Whitney *U* test in the validation phase (GraphPad Prism software version 4.0, GraphPad, Software Inc, San Diego, California, USA). The difference in time to recurrence between CK20-positive pN0 CRC patients with *VEGF-A* mRNA, *TCF20* mRNA and *CHD4* mRNA copy numbers that were ‘above’ (>) their respective median copy numbers, and those that were ‘below-or-equal’ (≤) their respective median values, was analyzed by the log-rank test. Multivariate regression (Cox proportional hazards regression) was used to analyze whether time to recurrence was correlated with the clinico-histopathological factors (Statistical Package for the Social Sciences Version 12.0 software, SPSS Inc., Chicago, IL., USA). A *P*-value < 0.05 was considered to be statistically significant.
